# Fructose Diet-Associated Molecular Alterations in Hypothalamus of Adolescent Rats: A Proteomic Approach

**DOI:** 10.3390/nu15020475

**Published:** 2023-01-16

**Authors:** Chiara D’Ambrosio, Luisa Cigliano, Arianna Mazzoli, Monica Matuozzo, Martina Nazzaro, Andrea Scaloni, Susanna Iossa, Maria Stefania Spagnuolo

**Affiliations:** 1 Institute for the Animal Production System in the Mediterranean Environment, National Research Council, 80055 Portici, Italy; 2 Department of Biology, University of Naples Federico II, 80121 Naples, Italy

**Keywords:** proteomics, hypothalamus, fructose-rich diet, adolescence, mitochondria, inflammation

## Abstract

Background: The enhanced consumption of fructose as added sugar represents a major health concern. Due to the complexity and multiplicity of hypothalamic functions, we aim to point out early molecular alterations triggered by a sugar-rich diet throughout adolescence, and to verify their persistence until the young adulthood phase. Methods: Thirty days old rats received a high-fructose or control diet for 3 weeks. At the end of the experimental period, treated animals were switched to the control diet for further 3 weeks, and then analyzed in comparison with those that were fed the control diet for the entire experimental period. Results: Quantitative proteomics identified 19 differentially represented proteins, between control and fructose-fed groups, belonging to intermediate filament cytoskeleton, neurofilament, pore complex and mitochondrial respiratory chain complexes. Western blotting analysis confirmed proteomic data, evidencing a decreased abundance of mitochondrial respiratory complexes and voltage-dependent anion channel 1, the coregulator of mitochondrial biogenesis PGC-1α, and the protein subunit of neurofilaments α-internexin in fructose-fed rats. Diet-associated hypothalamic inflammation was also detected. Finally, the amount of brain-derived neurotrophic factor and its high-affinity receptor TrkB, as well as of synaptophysin, synaptotagmin, and post-synaptic protein PSD-95 was reduced in sugar-fed rats. Notably, deregulated levels of all proteins were fully rescued after switching to the control diet. Conclusions: A short-term fructose-rich diet in adolescent rats induces hypothalamic inflammation and highly affects mitochondrial and cytoskeletal compartments, as well as the level of specific markers of brain function; above-reported effects are reverted after switching animals to the control diet.

## 1. Introduction

The crucial role of nutrition for cerebral health and the impact of dietary habits on brain structure and function has been long and far recognized [1,2,3]. To date, the increased consumption of fructose as added sugar in many types of drinks and processed foods [4,5], especially among young people [6], represents a major health concern. High fructose intake has been pointed out as the possible culprit for the raised incidence of chronic pathologies [7,8,9,10], and fructose over-consumption was shown to be associated with the over-activation of its cerebral metabolism [4], which was proposed to negatively impact on the whole brain physiology and cognitive function [4,11]. Notably, we previously reported that a short-term fructose-rich diet induces hippocampal mitochondrial dysfunction, oxidative stress, and neuroinflammation in young rats [12,13], as well as the imbalance of redox status, autophagic mechanisms and synaptic markers in the frontal cortex of both adult and young rats [14]. Further, studies in animal models have revealed that high-fructose diets impair hippocampal functions during childhood and adolescence [15,16,17,18,19], which represent critical phases of neurodevelopment.

The hypothalamus plays a key role in maintaining whole-body homeostasis [20,21]. Long-term fructose overfeeding was reported to alter the hypothalamic-pituitary-adrenal axis, thus causing glucocorticoid increase in peri-adolescent rats [22]. Further, fructose overconsumption was associated with impairment of hypothalamic insulin signaling [23,24], oxidative stress [24,25], and inflammation [23,26,27]; it was proposed that fructose-driven perturbations of hypothalamic function may alter the potential for satiety, thereby enhancing the possibility of developing obesity [28].

Data currently available on hypothalamic dysfunctions related to a high-fructose diet essentially refer to the effects of long-term sugar feeding, while information on corresponding alterations associated with short-term dietary treatment, particularly in the critical period of adolescence, is still lacking. Due to the complexity and multiplicity of hypothalamic functions, there is also the need for a holistic characterization aimed at unveiling the general picture of hypothalamic dysfunctions associated with a high-fructose diet. To fill this gap, we investigated adolescent rats fed a fructose-rich or control diet, for 3 weeks. To verify whether the fructose-driven changes persist after the switch to a control diet, half of the rats from both animal groups were then assigned to the control diet for additional 3 weeks until the young adulthood phase. Quantitative proteomics on hypothalamic extracts of all animal groups was used to identify molecular alterations triggered by a fructose-rich diet and to obtain insights into the relationship between sugar feeding and possible dysfunctions of the hypothalamus.

## 2. Materials and Methods

### 2.1. Materials

Bovine serum albumin fraction V (BSA), prestained protein ladder, salts, and buffers were purchased from DelTek (Naples, Italy). Rabbit anti-human haptoglobin IgG was from Sigma-Aldrich (St. Louis, MO, USA). Fuji Super RX 100 film, developer, and fixer were purchased by Laboratorio Elettronico di Precisione (Naples, Italy). BCA Protein assay kit, TMT Label Reagent Set, Pierce ™ High pH Reversed-Phase Peptide fractionation kit, Acclaim PepMap TM RSLC C18 column were from Thermo-Fisher Scientific (Waltham, MI, USA).

### 2.2. Experimental Design

**Figure 1:**
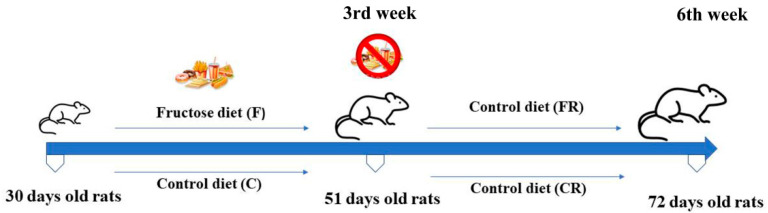
Experimental design.

Just weaned male Wistar rats (30 days old; Charles River; Calco, Como, Italy) were used. Housing, treatments, and euthanasia of animals were carried out as previously reported [13]. The rats were randomly assigned to two experimental groups, one fed a fructose-rich diet (F group; N = 16), and the other one fed a control diet (C group; N = 16) for 3 weeks. At the end of treatment, half of the rats from each group (C = 8; F = 8; 51 days old) were euthanized. Sugar-fed and control-fed rats were then assigned to the control diet for additional 3 weeks (FR group, N = 8; CR group, N = 8; respectively) until the young adulthood phase (72 days old). The experimental design is reported in Figure 1.

Thirty days-old rats (just weaned animals) received a control (C; N = 16) or fructose-rich (F; N = 16) diet for three weeks, until the adolescence phase (51 days old rats). In the 3rd week eight rats from each group were euthanized. Eight animals of both fructose-fed and control groups were fed the control diet (FR, N = 8; CR, N = 8) for further three weeks, until young adulthood (6th week).

The control and the fructose-rich diet were isocaloric, as they differ only in terms of the qualitative content of carbohydrates. Indeed, as shown in Table 1, the control diet contains starch instead of fructose. The here used control diet can be considered a glucose-containing diet, as starch digestion to glucose is very rapid [29,30].

Animals were then euthanized, brains were removed and placed on an ice-cooled glass plate putting the face down the cortex. For dissecting the hypothalamus from each brain incisions were made along these margins: laterally 2 mm of the third ventricle, dorsally 2 mm starting from the base, rostro caudally from the optic chiasm to the posterior side of the mammillary bodies. Samples were then snap-frozen in liquid nitrogen and stored at −80 °C for further analyses.

### 2.3. Protein Extraction

Proteins were extracted by homogenizing aliquots of the hypothalamus (about 40 mg) in six volumes (*w*/*v*) of cold RIPA buffer [31]. The protein concentration of each homogenate was evaluated spectrophotometrically, using a commercial colorimetric kit (Bio-Rad, Hercules, CA, USA). Protein extracts were used for proteomic investigations, ELISA, and western blotting analysis.

### 2.4. Proteomic Analysis

For quantitative proteomic analysis, the protein concentration of pooled samples was quantified with the BCA Protein assay kit, as specified by the manufacturer. Peptide preparation was carried out as previously reported [32]. The resulting peptides from each protein sample were tagged with the TMT Label Reagent Set according to the matching C-TMT6-126, CR-TMT6-127, F-TMT6-128, FR-TMT6-129, at 25 °C, in agreement to manufacturer’s instructions.

The analysis of TMT-labelled peptide fractions was carried out in triplicate on a nanoLC-ESI-Q-Orbitrap-MS/MS platform (Thermo Fisher Scientific, Waltham, MI, USA), as previously published [32].

All MS and MS/MS raw data files per sample were merged for protein identification and relative protein quantification into ProteomeDiscoverer 2.4 software (Thermo Scientific), enabling the database search by Mascot algorithm v. 2.4.2 (Matrix Science, London, UK) using the following criteria: UniProtKB protein database (*Rattus norvegicus*, 36206 protein sequences 11/2021). The mass spectrometry-based proteomic data have been deposited to the ProteomeXchange Consortium via the PRIDE partner repository with the dataset identifier PXD038834.

Functional enrichment analysis including GO and Kyoto Encyclopedia of Genes and Genomes (KEGG) pathway was performed by using EnrichR (https://amp.pharm.mssm.edu/Enrichr, accessed on 1 December 2022) and FunRich 3.1.3 (http://www.funrich.org, accessed on 25 November 2022). STRING (https://string-db.org, accessed on 5 August 2022) was used to visualize and integrate complex networks of proteomic data.

### 2.5. Western Blotting

Hypothalamic proteins (30 µg) were fractionated by denaturing and reducing electrophoresis [33] by 12.5% (to titrate glial fibrillary acidic protein (GFAP), synaptophysin, brain-derived neurotrophic factor (BDNF), voltage-dependent anion-selective channel 1 (VDAC-1), PTEN-induced putative kinase 1 (PINK-1), adiponectin, respiratory mitochondrial complexes I-V (OXPHOS)) or 10% (nuclear factor kappa-light-chain-enhancer of activated B cells, NFkB; post-synaptic density protein 95 (PSD-95), synaptotagmin I, peroxisome proliferator-activated receptor gamma coactivator 1-alpha [PGC-1α], tropomyosin receptor kinase B (TrkB), α-internexin) polyacrylamide gels. Protein blotting onto PVDF or nitrocellulose membrane (GE Healthcare; Milan, Italy), washing, and blocking steps were carried out according to previously published procedures [12,34].

The membranes were treated with primary antibodies (overnight, at 4 °C), and then incubated (1 h, at 37 °C) with the appropriate peroxidase-conjugated secondary antibodies. The specific dilution of each antibody is shown in Table 2. In particular, as the accurate quantification of each mitochondrial complex requires the use of different dilutions of secondary antibody for optimizing band intensities [35], it was used GAM-HRP IgG diluted 1:100,000 for the detection of complex I, 1:80,000 for complex II, 1:70,000 for complex IV, and 1:200,000 for complexes V and II.

After detection of each antigen, the membranes were stripped [36] and incubated (overnight, 4 °C) with mouse anti-β-actin IgG (1:1000 in 0.25% *v*/*v* non-fat milk) followed by GAM-HRP IgG (1:30,000 in 0.25% *v*/*v* non-fat milk; 1 h, 37 °C), in order to reveal β-actin, which was used as a loading control. Signal detection was carried out using the Excellent Chemiluminescent Kit Westar Antares (Cyanagen s.r.l., Bologna, Italy). Densitometric analysis of chemidoc or digital images of X-ray films exposed to immunostained membranes was performed with Un-Scan-It gel software (Silk Scientific, Orem, UT, USA).

### 2.6. Analysis of Tumor Necrosis Factor Alpha (TNF-alpha) and Interleukin 6 (IL-6)

TNF-alpha and IL-6 concentrations were evaluated by sandwich ELISA with the DuoSet ELISA kit (R&D, DBA Italia, Segrate, MI, Italy), following the manufacturer’s instructions. Hypothalamic homogenates were diluted 1:25 in the assay [37], and data are expressed as pg per mg of total proteins.

### 2.7. Evaluation of Nitro-Tyrosine and Haptoglobin (Hpt)

Nitro-tyrosine (N-Tyr) concentration was measured by ELISA in hypothalamic samples diluted 1:1500, 1:3000, and 1:6000 with coating buffer (7 mM Na_2_CO_3_, 17 mM NaHCO_3_, 1.5 mM NaN_3_, pH 9.6), essentially according to a previously published procedure [13]. Results are expressed as OD per mg of total proteins.

Haptoglobin (Hpt) was titrated by ELISA, in samples diluted 1: 2000, 1:8000, 1:25,000 with coating buffer, according to [38].

### 2.8. Statistical Analysis

Data are reported as mean values ± SEM. Normal distribution of data was verified with the GraphPad Prism 9.3.1 program (GraphPad Software, San Diego, CA, USA); the same software was used to perform one-way ANOVA followed by Bonferroni post-test. *p* < 0.05 was assumed as significant in the reported analyses.

## 3. Results

### 3.1. Identification of Fructose-Induced Hypothalamic Changes by Proteomic Analysis

**Figure 2:**
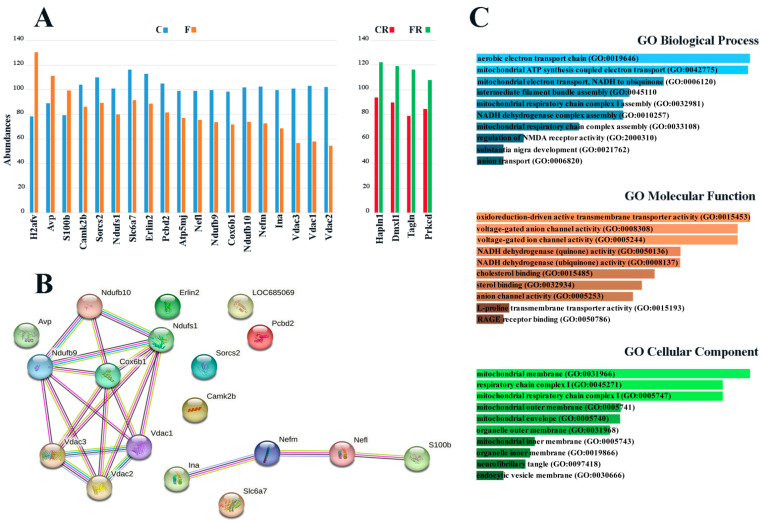
Proteomic analysis of rat hypothalamus. (**A**) Proteins showing quantitative changes in the comparison of fructose-fed adolescent (F, orange) versus control adolescent (C, light blue) rats, and young-adult fructose-rescued (FR, green) versus young-adult control rescued (CR, red) rats. Differentially represented proteins are indicated using corresponding gene names. (**B**) STRING analysis of differentially represented proteins present in F versus C fed rats. Functional protein associations were based on data recorded for *R. norvegicus*. Only medium-confidence interactions (0.4) are shown. Sorcs2, sortilin-related VPS10 domain containing receptor 2; Ndufs1, NADH-ubiquinone oxidoreductase 75 kDa subunit, mitochondrial; Atp5mpl (Atp5mj), mitochondrial membrane ATP synthase F (1) F (0); Camk2b, calcium/calmodulin-dependent protein kinase type II, subunit beta; S100b, protein S100-B; Ndufb10, NADH:ubiquinone oxidoreductase subunit B10; Vdac2, voltage-dependent anion-selective channel protein 2; Vdac1, voltage-dependent anion-selective channel protein 1; Ndufb9, NADH dehydrogenase (ubiquinone) 1 beta subcomplex 9; Slc6a7, solute carrier family 6 (neurotransmitter transporter, l-proline) member 7; Avp, vasopressin-neurophysin 2-copeptin; Nefl, neurofilament, light polypeptide; Erlin2, ER lipid raft associated 2; Cox6b1, cytochrome c oxidase, subunit VIb polypeptide 1; Vdac3, voltage-dependent anion-selective channel protein 3; LOC685069, H2A histone family, member V; Ina, internexin neuronal intermediate filament protein, alpha; Nefm, neurofilament, medium polypeptide; Pcbd2, 4a-hydroxytetrahydrobiopterin dehydratase. (**C**) Functional enrichment analysis of differentially represented proteins in F versus C fed rats. GO terms for biological process (upper panel), molecular function (middle panel) and cellular component (lower panel) are reported.

Quantitative protein evaluation performed in the four experimental groups according to a Tandem Mass Tag (TMT)-based proteomic approach [39], followed by bioinformatic analysis, allowed the identification and quantification of 2521 unique protein entries in rat hypothalamus. Based on precise and accurate quantitation characteristics of the TMT-based proteomic approach, as determined in previous comparative studies on label-free and label-based procedures [40,41], only proteins with a concomitant fold change value > 1.2 and an abundance ratio *p*-value < 0.05 were considered as differentially represented. Accordingly, three proteins resulted to be over-represented, and sixteen were under-represented in the F group, with respect to the C group (Figure 2A, Supplementary Materials Figures S1 and S2); on the other hand, four proteins were over-represented in the FR group with respect to CR (Figure 2A; Supplementary Materials Figure S2). Slight protein quantitative changes observed in F compared to the FR group, and in C compared to the CR group, were associated with youth to adolescence physiological changes, and thus were not considered in this study. Detailed quantitative proteomic data are reported in Supplementary Materials Table S1.

Differentially represented proteins in groups F and C were analyzed with the STRING tool, using as a template the UniProtKB database of *R. norvegicus*; this allowed for predicting a functional protein association map, which was characterized by two ramified networks including 19 nodes and linking together 11 components, plus eight non-associated species (Figure 2B). On the other hand, functional analysis of differentially represented components according to KEGG metabolic pathway classification highlighted selective enrichment of proteins mainly involved in cholesterol metabolism, ferroptosis, oxidative phosphorylation, Parkinson’s disease, and necroptosis. Several deregulated proteins belong to the GO Cellular Component Mitochondrial membrane, Mitochondrial respiratory chain complex I, and Mitochondrial outer membrane (Figure 2C).

### 3.2. Reduced Amount of Mitochondrial Respiratory Complexes, PGC-1α and VDAC-1 and Higher Level of PINK-1 in Hypothalamus of Fructose-Fed Adolescent Rats

**Figure 3:**
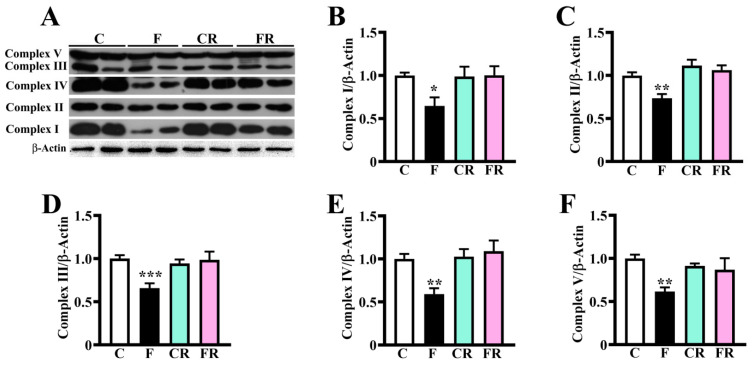
Mitochondrial respiratory complexes amount in rat hypothalamus. The amount of mitochondrial complex I, II, III, IV, and V was measured in protein extracts from the hypothalamus of control adolescent (C), fructose-fed adolescent (F), young-adult control rescued (CR), young-adult fructose-rescued (FR) rats. Samples were analyzed by 12.5% SDS-PAGE and western blotting. After the detection of immunocomplexes (by mouse anti-OXPHOS and GAM-HRP IgGs) the membrane was stripped and treated with anti-β-actin IgG as the loading control. (**A**) Representative western blotting of respiratory complexes. Panels B-E show results from densitometric analysis. (**B**) Complex I amount relative β-actin; (**C**) Complex II amount relative to β-actin; (**D**) Complex III amount relative to β-actin; (**E**) Complex IV amount relative to β-actin; (**F**) Complex IV amount relative to β-actin; (**F**): Complex V amount relative to β-actin. Data shown are reported as means ± SEM of eight animals from each group * *p* < 0.05, ** *p* < 0.01, *** *p* < 0.001 versus C (one-way Anova followed by Bonferroni post-test).

Proteomic investigation revealed that fructose feeding determined a down-representation of specific proteins belonging to mitochondrial respiratory complexes I (Ndufs1, Ndufb9, and Ndufb10), IV (Cox6b1), and V (Atp5mj) (Figure 2A). With the aim to validate these diet-induced alterations, we further assessed by western blotting the abundance of complexes I–V. In line with proteomic data, the amount of all respiratory complexes was lower in fructose-fed than in control rats (*p* < 0.01; Figure 3), strongly suggesting that a short-term fructose diet generates early quantitative alterations of the oxidative phosphorylation system.

**Figure 4:**
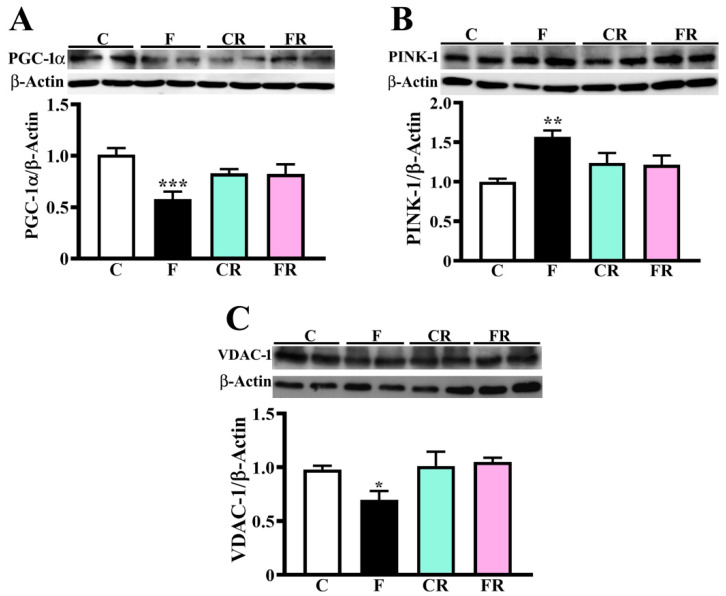
PGC-1α, PINK-1 and VDAC-1 levels in rat hypothalamus. PGC-1α (**A**), PINK-1 (**B**), and VDAC-1 (**C**) were quantified in protein extracts from the hypothalamus of control adolescent (C), fructose-fed adolescent (F), young-adult control rescued (CR), young-adult fructose-rescued (FR) rats. Samples were analyzed by 10% (PGC-1α) or 12.5% (PINK-1, VDAC-1) SDS-PAGE and western blotting. After detection of immunocomplexes [by rabbit anti-PGC-1α and GAR-HRP IgGs (**A**), or mouse anti-PINK-1 and GAM-HRP IgGs (**B**), or mouse anti-VDAC-1 and GAM-HRP IgGs (**C**)], the membranes were stripped and treated with anti-β-actin as loading control. Data shown are means ± SEM of eight animals from each group * *p* < 0.05, ** *p* < 0.01, *** *p* < 0.001 versus C (one-way Anova followed by Bonferroni post-test).

Based on these evidences, we measured the protein levels of PGC-1α, which is a critical coregulator of transcription factors participating in the regulation of mitochondrial biogenesis and respiration [42,43,44], cellular metabolism [45], and detoxification of reactive oxygen species (ROS) produced by mitochondrial respiration [46,47], thus exerting a global positive impact on oxidative metabolism. The amount of PGC-1α was lower in F than the control group (*p* < 0.001; Figure 4A), in line with results on corresponding respiratory complexes. Since the decreased abundance of respiratory complexes and PGC-1α suggested a whole damage of the mitochondrial compartment, we further investigated whether fructose feeding affects the levels of PINK-1, which is a protein involved in the modulation of mitophagy [48]. A significant increase of this protein was evidenced in the F group (*p* < 0.01; Figure 4B), strongly supporting the hypothesis that the fructose diet was also associated with mitophagy activation and decreased mitochondrial biogenesis.

Proteomic results also revealed a significant decrease in the abundance of three voltage-dependent anion channel (VDAC) proteins (Figure 2A), namely VDAC-1, 2 and 3, which are placed in the outer mitochondrial membrane, and whose alteration is known to contribute to pathological states [49]. As shown in Figure 4C, VDAC-1 levels were reduced in fructose-fed rats (*p* < 0.05), confirming proteomic data, and also suggesting the occurrence of an impairment in the communication between the mitochondrial matrix and the cytosol, and a possible alteration of the mitochondrial functions.

The switch to the control diet determined the rescue of all the above-described protein changes, as demonstrated by the finding of no difference in the levels of these components was observed between CR and FR rats (Figure 2B, Figure 3 and Figure 4).

### 3.3. Increased Levels of Inflammatory and Oxidative Stress Markers in Hypothalamus of Fructose-Fed Adolescent Rats

**Figure 5:**
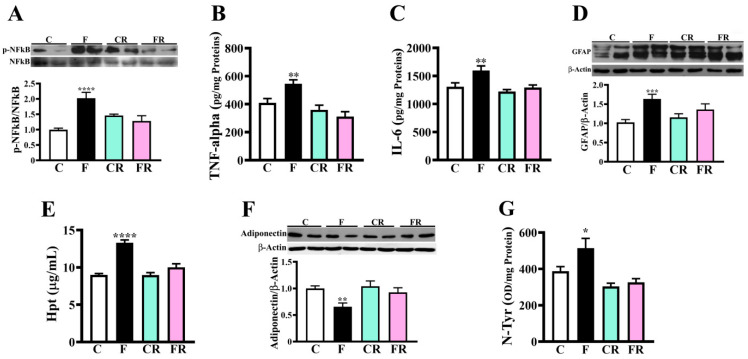
Evaluation of inflammatory and oxidative status in rat hypothalamus. (**A**) p-NFkB/NFkB ratio (representative western blotting and densitometric analysis). Samples were analyzed by 10% SDS-PAGE and western blotting. After rivelation of the immunocomplexes (by mouse anti-phosphoNFkB and GAM-HRP IgGs), the membrane was stripped and treated with mouse anti-NFkB and GAM-HRP IgGs. The amount of phosphorylated NFkB was expressed relative to the total NFkB level. Data shown are means ± SEM of eight animals from each group. (**B**) TNF-alpha and (**C**) IL-6 amount was titrated by sandwich ELISA on samples diluted 1:25 and following the manufacturer’s instructions. Data are expressed as pg per mg of total proteins and reported as means ± SEM of eight different rats from each experimental group. (**D**) GFAP amount (representative western blotting and densitometric analysis). Samples were analyzed by 12.5% SDS-PAGE and western blotting. After the rivelation of immunocomplexes (by rabbit anti-GFAP and GAR-HRP IgGs), the membrane was stripped and re-incubated with anti-β-actin. Data shown are means ± SEM of eight animals from each group. (**E**) Hpt level was titrated by ELISA in samples diluted 1: 2000, 1:8000 and 1:25,000. Immunodetection was carried out with rabbit anti-Hpt and GAR-HRP IgGs. Data are means ± SEM of eight animals from each group. (**F**) Adiponectin amount (representative western blotting and densitometric analysis). Samples were analyzed by 12.5% SDS-PAGE and western blotting. After rivelation of the immunocomplexes (by rabbit anti-adiponectin and GAR-HRP IgGs), the membrane was stripped and re-incubated with anti-β-actin. Data shown are means ± SEM of eight animals from each group. (**G**) N-Tyr amount was titrated by ELISA on samples diluted 1:1500, 1:3000 and 1:6000. Immunodetection was carried out with rabbit anti-N-Tyr and GAR-HRP IgGs. Data are expressed as OD per mg of total proteins and reported as means ± SEM of eight different rats from each experimental group. Control (C), fructose-fed (F), control rescued (CR), and fructose-rescued (FR) rats. * *p* < 0.05, ** *p* < 0.01, *** *p* < 0.001; **** *p* < 0.0001 versus C (one-way Anova followed by Bonferroni post-test).

Proteomic results also revealed a significant fructose-associated increase of S-100B protein (Figure 2A), which is known to participate in the induction of the inflammatory cascade, astrocyte activation, and oxidative stress [50,51,52]. Therefore, we evaluated NF-kB activation and the levels of TNF-alpha, IL-6, and GFAP, to verify whether the increase of S-100B in fructose-fed rats is associated with a rise in hypothalamic inflammation. The degree of NFkB phosphorylation was measured as the ratio between phosphorylated and total NFkB (p-NFkB/NFkB), and then used as a marker of the activation of the NFkB pro-inflammatory signaling pathway. As shown in Figure 5A, the p-NFkB/NFkB ratio was found higher in fructose-fed than in control rats (*p* < 0.0001). Further, both TNF-alpha and IL-6 concentrations were higher in F than in the C group (*p* < 0.01; Figure 5B,C). Similarly, higher amounts of GFAP (*p* < 0.001; Figure 5D) and Hpt (*p* < 0.0001; Figure 5E), an inflammatory marker sensitive to nutritional status [37,38], were detected in the hypothalamus of F rats. Altogether, these results strongly suggested that the increase of S-100B levels observed as a result of short-term high-fructose feeding might be responsible for NF-kB activation in the hypothalamus, which in turn promotes inflammatory cytokine production and astrocyte activation, leading to a general condition of inflammation therein.

Finally, the concentration of adiponectin, which is an adipokine exerting antioxidant, anti-inflammatory, and neuroprotective effect [53,54], was lower in sugar-fed than in control rats (*p* < 0.01; Figure 5F). Similarly, plasma levels of adiponectin were found to decrease in F rats with respect to C ones (*p* < 0.01; Supplementary Materials Figure S3), with persistent lower levels in FR compared to the CR group (*p* < 0.05; Supplementary Materials Figure S3). In line with the observed reduction in F rats of adiponectin, fructose feeding was also associated with the increased concentration of N-Tyr (*p* < 0.05; Figure 5G), which is a marker of protein oxidation induced by peroxynitrite [55].

The switch to the control diet determined the rescue of all the above-reported protein changes, as demonstrated by the finding of no difference in the levels of these components between CR and FR rats (Figure 2B and Figure 5).

### 3.4. Decreased Amount of Neuronal Intermediate Filaments, BDNF, and Synaptic Markers in Hypothalamus of Fructose-Fed Adolescent Rats

**Figure 6:**
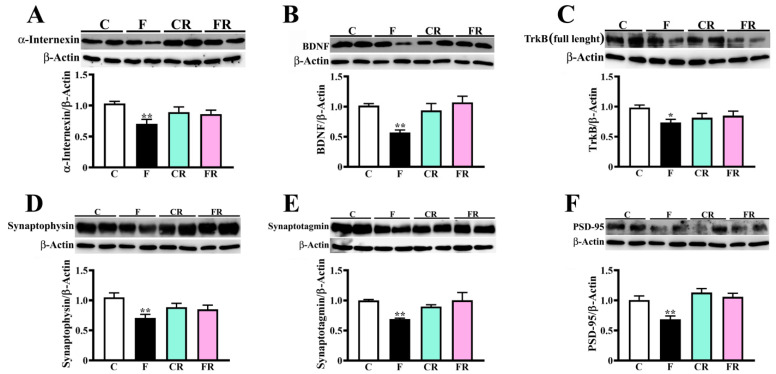
Evaluation of α-internexin, BDNF, TrkB, and synaptic proteins in rat hypothalamus. (**A**) α-internexin, (**B**) BDNF, (**C**) full-length TrkB isoform, (**D**) synaptophysin, (**E**) synaptotagmin, and (**F**) PSD-95 were measured in protein extracts from the hypothalamus of control adolescent (C), fructose-fed adolescent (F), young-adult control rescued (CR), young-adult fructose-rescued (FR) rats. Samples were analyzed by 10% (α-internexin, TrkB, synaptotagmin, PSD-95) or 12.5% (BDNF, synaptophysin) SDS-PAGE and western blotting. Immunocomplexes were detected by rabbit mouse anti-α-internexin and GAM-HRP IgGs (**A**), or rabbit anti-BDNF (**B**), rabbit anti-TrkB (**C**), rabbit anti-synaptophysin (**D**), rabbit anti-synaptotagmin (**E**), rabbit anti-PSD-95 (**F**) and GAR-HRP IgGs. After detection, the membranes were stripped and treated with anti- β-actin. Means ± SEM of eight animals from each experimental group are shown. * *p* < 0.05, ** *p* < 0.01 versus C (one-way Anova followed by Bonferroni post-test).

Proteomic analysis (Figure 2A) also showed a significant reduction of the abundance of neurofilament light protein, neurofilament medium protein, and alpha-internexin, which are subunits of neuronal intermediate filaments, namely neurofilaments, in fructose-fed rats compared to control rats. The amount of α-internexin was also measured by western blotting and this approach confirmed a significant decrease in protein levels in fructose-fed rats (*p* < 0.01; Figure 6A), with respect to control, validating proteomic data. As neurofilament proteins and α-internexin are the major components of neurons’ cytoskeleton and their lack has already been related to neuronal loss [56], we may hypothesize that short-term fructose feeding might cause damage to nerve cells.

BDNF signaling participates in the modulation of different neurophysiological processes [57], such as synaptic function, dendritic spine maturation, and stabilization. Accordingly, we investigated whether the amounts of this neurotrophin in hypothalamic tissues are affected by fructose feeding. We observed that BDNF levels were halved in sugar-fed rats (*p* < 0.01; Figure 6B). Further, the amount of the full-length TrkB isoform, which is the high-affinity receptor of BDNF, was significantly lower in the F than in the C group (*p* < 0.05; Figure 6C), suggesting that short-term sugar feeding negatively impacts both BDNF production and signaling. These results are also in good agreement with proteomic data reporting a lower abundance of Sorcs2 protein in the F group compared with C one (Figure 2A). As Sorcs2 traffics TrkB to the postsynaptic membrane and interacts with TrkB, forming a complex that is essential for BDNF signalling [58], we may hypothesize that the observed fructose-associated decrease of SorCS2 should contribute to the alteration of neurotrophin functions.

Then, we evaluated the amount of presynaptic proteins synaptophysin and synaptotagmin, as well as postsynaptic protein PSD-95. High-fructose diet was associated with a significant reduction of both synaptophysin and synaptotagmin (*p* < 0.01 r; Figure 6D,E), as well as of PSD-95 (*p* < 0.01; Figure 5F).

The switch to the control diet fully rescued all above-reported protein alterations, as demonstrated by the finding of no difference in the levels of these components was detected between CR and FR rats (Figure 2B and Figure 6).

## 4. Discussion

High-fructose intake has been reported to have numerous unhealthy consequences within the spectrum of metabolic syndromes, impacting genes related to several metabolic pathologies, and correlating with increasing incidence of neurologic diseases [5,59,60,61]. Several studies on rodent models have shown that fructose and glucose modulate in a different manner the cerebral pathways regulating appetite and feeding behavior [62]. As a matter of fact, central administration of fructose stimulates feeding, while glucose induces satiety in rodents [63,64]; it was also proposed that fructose-containing diets, as provoking smaller raises of insulin and leptin secretion compared to glucose [65,66], exert a lower inhibition on orexigenic neurons [67]. Long-term fructose feeding was shown to be associated with a high preference and motivation for the sugar-based diet [68], to elicit leptin resistance in rats, due to a defective transport of the protein through the blood-brain barrier [69,70], and also to determine upregulated expression of neuropeptide Y (NPY) [70]. On the other hand, a short-term fructose diet stimulates the production of ghrelin, and concomitantly down-regulates the satiety signal peptide YY3-36 and the orexigenic NPY, determining a cooperative effect in the modulation of appetite and food intake [71].

Since the hypothalamus is the master center for control of brain and body homeostasis, it was recently proposed that the effects of fructose on hypothalamic metabolism might play a key role in triggering metabolic disorders, which in turn may determine neurological effects, ultimately affecting brain functions and behavior [5,72]. However, current knowledge mostly refers to prolonged dietary treatments, whereas no information is available about the effects of short-term fructose feeding. Our challenge was to identify molecular mechanisms early influenced by sugar feeding in the hypothalamus through the investigation of corresponding protein changes associated with a short-term fructose-rich diet, focusing on a critical phase of growth, namely adolescence.

The proteomic analysis allowed us to portray early quantitative molecular changes associated with fructose consumption, evidencing 19 proteins differentially represented between control and treated rats. A strong alteration was observed in the amount of specific mitochondrial proteins, such as the components of respiratory complexes I (Ndufs1, Ndufb9, and Ndufb10), IV (Cox6b1), and V (ATP5MJ), whose levels were reduced by about 30%. These proteins are part of complexes placed in the inner mitochondrial membrane and are responsible for oxidative phosphorylation as well as of ATP production [73]. The western blotting analysis demonstrated a significant reduction in the level of protein components of complex V (ATP5A), IV (MTCO1), and I (NDUFB8), validating proteomic results, and also showed a significant decrease of specific elements of complexes II and III. Considering that complex I protein NDUFB8 and complex IV protein MTCO1 are widely used as biomarkers of mitochondrial content [74,75], the finding of a quantitative reduction of components of all mitochondrial respiratory complexes was suggestive of a deleterious effect of a short-term high-fructose diet on mitochondrial biogenesis. Our hypothesis of a fructose-associated alteration of mitochondrial abundance was supported by the finding of the decreased abundance of the biogenesis-related protein PGC-1α, and higher amounts of the mitophagy marker PINK-1 [48,76] in the hypothalamus of treated rats. In this context, although proteomic investigation did not reveal changes in the abundance of proteins belonging to the mitochondrial fission and fusion machinery (data not shown), future studies on mitochondrial proteome and respiration might contribute to better clarifying fructose effect on mitochondria dynamics and quality control mechanisms, which are essential for maintaining their proper environment and functioning.

The proteomic analysis also evidenced a strong decrease in the abundance of VDAC-1, VDAC-2, and VDAC-3 in short-term high-fructose-fed rats, which was confirmed in the case of VDAC-1 by immunoblotting. VDAC proteins are mitochondrial porins localized on the outer membrane, involved in the exchange of molecules between the mitochondrial matrix and cytoplasm, as well as in the docking of cytosolic and mitochondrial proteins [49]. In particular, VDAC-1 is considered a gatekeeper for mitochondrial functions, and its alteration was reported to contribute to the pathogenesis of several diseases [49]. Since VDAC-1 acts as a hub protein modulating the integration between mitochondrial and other cellular activities [77], our results on the lower abundance of VDAC-1 in fructose-fed rats led us to hypothesize that a sugar-rich diet negatively impacts the whole mitochondrial compartment and, likely, on hypothalamic cells functions. The observed compromising of the mitochondrial compartment following the fructose feeding represents a deleterious culprit for hypothalamic functions but also an alarm of the possible predisposition to the development of degenerative phenomena in the long-term, as mitochondrial dysfunction and impaired organelle dynamics are linked to neurodegenerative and metabolic diseases [78,79,80]. We previously reported a fructose-induced impairment of complex II activity in the hippocampus of adolescent rats [13]. Therefore, an interesting goal of future research will be investigating whether short-term fructose feeding alters hypothalamic mitochondrial respiratory functions by evaluating possible dysfunction in electron transport, ADP phosphorylation, and leak respiration across the inner mitochondrial membrane.

Interestingly, proteomic experiments also highlighted increased levels of S-100B protein in high-fructose-fed rats, thus hinting at an inflammatory status in these animals. Indeed, the S-100B protein, when overproduced by activated glia, acts as a pro-inflammatory cytokine, and contributes to neuroinflammation heightening and neuronal dysfunction [50]. Hence, this protein promotes the migration and activation of microglia [81], also inducing TNF-alpha expression therein [52,82] and induces an autocrine loop in astrocytes, which results in the stimulation of IL-6 and TNF-alpha secretion [51]. In agreement with the detected increase of S-100B protein in the hypothalamus of high-fructose-fed rats, we observed an enhanced NF-kB activation, higher levels of the astrocytic marker GFAP, and the rise of two inflammatory cytokines, TNF-α and IL-6, as well as of Hpt. These results led us to hypothesize that the fructose-associated gain of S-100B is a trigger event driving hypothalamic inflammation. It is worth mentioning that hypothalamic inflammation is considered a potential trigger for the deregulation of mechanisms involved in food control and energy metabolism [28,83,84]. As a matter of fact, a long-term fructose diet was reported to induce increased IL-6 and TNF-alpha expression in mice hypothalamus, together with down-regulation of the expression of both the anorexigenic proopio-melanocortin (POMC) and the orexigenic NPY [85]. As above mentioned, several animal studies demonstrated the orexigenic effect of fructose [28]. Indeed, long-term fructose feeding in rats was associated with increased expression of the NPY gene [26,70,86], reduced POMC expression and impairment in the melanocortin system [26], and augmented gene expression of the agouti-related peptide [70,86]. However, it was pointed out that results regarding fructose effects on food intake and energy metabolism modulation are often contrasting and essentially depend on the animal model, intake doses, mode, and duration of administration [62]. In our experimental model, dietary treatment was not associated with changes in body weight and daily energy intake [87]. Accordingly, the proteomic investigation did not reveal POMC abundance changes (data not shown), maybe because of the young age of rats or the short duration of diet administration. Nevertheless, we cannot exclude that hypothalamic inflammation might be linked to alterations in the expression of orexigenic and/or anorexigenic genes; future analyses will be critical to clarify this issue.

The hypothesis of a diet-associated hypothalamic impairment was also supported by the finding, in fructose-fed rats, of decreased levels of adiponectin, a protein playing physiological functions in the brain, regulating synaptic plasticity [88], preventing oxidative stress and suppressing inflammatory cascade [89,90,91]. Hence, the decrease of adiponectin in high-fructose-fed rats might represent a further molecular contribution to hypothalamic inflammation, also depriving this region of protection against the pro-oxidant effect of inflammatory cytokines. Although recent studies demonstrated adiponectin expression in the hypothalamus of mice, beavers, or female pigs [92,93,94,95], we cannot exclude that adipocytes are the source of the protein we measured in the hypothalamus. Indeed, peripheral adiponectin can cross the blood-brain barrier reaching the brain [96,97,98]. Accordingly, we measured lower adiponectin levels in the plasma of sugar-fed rats. Further, as PGC-1α is also known to participate in the detoxification of ROS [46,47], the observed decrease of both this protein and adiponectin in fructose-fed rats was also suggestive of a condition of oxidative stress in the hypothalamus of these animals. Indeed, lower levels of N-Tyr corroborated the alteration of the redox balance in high-fructose-fed rats.

Proteomic results also revealed a decrease in the amount of three protein components (neurofilament light, NF-L; neurofilament medium, NF-M; alpha-internexin) of neurofilaments (NFs) in fructose-fed rats. In the case of alpha-internexin, these results were confirmed by western blotting. NFs play a key structural role in neurons [99,100], are essential for synaptic functions [101,102], and form cellular scaffolds involved in the docking and organization of synaptic vesicles, endosomes, and endoplasmic reticulum [103]. Because of the multiple key functions of NFs in neurons, the observed alteration of the cytoskeleton scaffold led us to suppose that the damaging effect of a high-fructose diet might also lead to a disruption of the neuronal network, with possible deleterious impact on corresponding functions. In addition to the above-mentioned results on NF-L, our proteomic analysis notably demonstrated a slight (about 18%) but significant decrease in fructose-fed rats of CaMKII2b, which is a neuronal isoform of CAMKII crucial for both growth and arborization of dendrites during the developmental phase [104]. This protein also promotes synapse and spine formation and elongation [105], controls dendritic morphology, neurite extension, and synapse number, and is essential for long-term plasticity, learning, and memory consolidation [106]. This finding further supports the hypothesis of a fructose-induced neuronal dysfunction, which could also affect synaptic functions.

In fructose-fed rats, we also observed a decreased amount of both BDNF and its high-affinity receptor TrkB, in line with data previously obtained in the prefrontal cortex of adult [14] and young [107] rats fed the same sugar-rich diet. In this context, proteomics also detected a concomitant decrease of SorCS2, which is a protein localized within synaptic vesicles and at the post-synaptic density of dendrites [58,108]. SorCS2 traffics TrkB to the postsynaptic membrane, and it was reported to interact with TrkB, forming a complex that is fundamental for several BDNF-dependent processes such as hippocampal long-term potentiation and changes in dendritic complexity and spine density [58]. As BDNF-TrkB signaling is crucial in supporting neuronal survival, differentiation, and growth, as well as synaptic transmission [57], the decrease of both neurotrophin and receptor corroborates the idea of a fructose-induced disruption of synaptic plasticity. In line with this hypothesis, we observed lower levels of synaptophysin, synaptotagmin, and post-synaptic density protein 95 (PSD-95) in the hypothalamus of F rats, compared to control ones. Since the TrkB receptor is associated with PSD-95, and BDNF/TrkB signaling increases both the recruitment of PSD-95 to synapses [109] and its localization at dendritic spines [110], the observed decrease of both BDNF and TrkB in F rats may be responsible for the decrement of PSD-95, confirming a fructose-associated impairment of BDNF-depending molecular mechanisms. The decreased amount of these synaptic markers in fructose-fed rats, together with the concomitant reduction of levels of BDNF, neurofilament proteins, and PGC-1α, which is known to participate in the regulation of brain plasticity by modulating spinogenesis and synaptogenesis [111], prompted us to suggest that short-term sugar feeding might negatively impact on synapse number, and may cause synaptic dysfunctions and impaired brain network activities.

## 5. Conclusions

To the best of our knowledge, this is the first study examining both the effect of a short-term fructose-rich diet on the hypothalamus, throughout adolescent development, and the possible persistence of sugar-induced changes until young adulthood, after returning to a balanced diet. Overall, the results here reported indicating that a diet rich in fructose, even if accomplished for short period, highly affects mitochondrial and cytoskeletal compartments, as well as the level of specific markers of brain function and plasticity. Interestingly, unlike what we observed in the frontal cortex of fructose-fed rats [107], the fructose-driven changes detected in the hypothalamus were fully rescued after switching to the control diet. Further studies comparing the effect of fructose and other sugars will be important to evaluate the possible use of alternative sugars for processed food.

## Figures and Tables

**Table 1:** Nutritional composition of diets.

Ingredients (g/100 g)	Control Diet	Fructose Diet
Standard Chow ^a^	50.5	50.5
Sunflower Oil	1.5	1.5
Casein	9.2	9.2
Alphacel	9.8	9.8
Cornstarch	20.4	-
Fructose	-	20.4
Water	6.4	6.4
AIN-76 mineral mix	1.6	1.6
AIN-76 vitamin mix	0.4	0.4
Choline	0.1	0.1
Methionine	0.1	0.1
Energy content and composition		
Gross Energy Density (kJ/g)	17.2	17.2
ME content (kJ/g) ^b^	11.1	11.1
Proteins (% ME)	29.0	29.0
Lipids (% ME)	10.6	10.6
Carbohydrates (% ME)	60.4	60.4
Of which:		
Fructose	-	30.0
Starch	52.8	22.8
Sugars	7.6	7.6

^a^ 4RF21, Mucedola, Italy. ^b^ Estimated by computation using the following values (kJ/g) for energy content: proteins = 16.736, lipids = 37.656, carbohydrates = 16.736. ME = metabolizable energy.

**Table 2:** Dilutions of primary and secondary antibodies used for Western blotting.

	Primary Antibody	Secondary Antibody
**GFAP**	Cell Signalling Technology; 1:1000 ^a^	GAR-HRP IgG; :100,000 ^f^
**Synaptophysin**	Merk-Millipore; 1:100,000 ^b^	GAR-HRP IgG; 1:35,000 ^a^
**Synaptotagmin I**	Cell Signalling Technology; 1:1000 ^c^	GAR-HRP IgG; :200,000 ^c^
**PSD-95**	Cell Signalling Technology; 1:1000 ^c^	GAR-HRP IgG; 1:60,000 ^c^
**BDNF**	Abcam, Cambridge, UK (EPR1292); 1:2000 ^d^	GAR-HRP IgG; 1:180,000 ^a^
**PGC-1α**	Merk-Millipore; 1:2000 ^b^	GAR-HRP IgG; 1:40,000 ^b^
**TrkB**	Santa Cruz Biotechnology; 1:2000 ^d^	GAR-HRP IgG; :100,000 ^d^
**VDAC 1**	Santa Cruz Biotechnology; 1:500 ^d^	GAM-HRP IgG; :50,000 ^b^
**α-internexin**	Santa Cruz Biotechnology; 1:500 ^d^	GAM-HRP IgG; :70,000 ^b^
**PINK1**	Santa Cruz Biotechnology; 1:500 ^d^	GAM-HRP IgG; 1:40,000 ^b^
**OXPHOS**	Abcam, Cambridge, UK; 1:400 ^b^	GAM-HRP IgG; 1:70,000–1:200,000 ^a^
**pNFkB**	Santa Cruz Biotechnology; 1:200 ^d^	GAM-HRP IgG; 1:50,000 ^b^
**NFkB**	Santa Cruz Biotechnology; 1:500 ^b^	GAM-HRP IgG; 1:15,000 ^b^
**β-Actin**	Sigma-Aldrich; 1:1000 ^e^	GAM-HRP IgG; 1:30,000 ^e^

GAR-HRP: Goat anti-rabbit Horseradish peroxidase-conjugated IgG (Immunoreagents, Raleigh, NC, USA). GAM-HRP: Goat anti-mouse Horseradish peroxidase-conjugated IgG (Immunoreagents, Raleigh, NC, USA). T-TBS: 130 mM NaCl, 20 mM Tris-HCl, 0.05% Tween, pH 7.4; ^a^ T-TBS containing 1% *v*/*v* non-fat milk; ^b^ T-TBS containing 3% *w*/*v* BSA; ^c^ T-TBS containing 3% *v*/*v* non-fat milk; ^d^ T-TBS containing 2% *w*/*v* BSA; ^e^ T-TBS containing 0.25% *v*/*v* non-fat milk.

## Data Availability

The mass spectrometry proteomics data have been deposited to the ProteomeXchange Consortium via the PRIDE partner repository with the dataset identifier PXD038834. All data supporting the findings of this study are available within the article and its supplementary information files.

## Supplementary Materials

The following supporting information can be downloaded at: https://www.mdpi.com/article/10.3390/nu15020475/s1. Table S1: Identification details of differentially represented proteins; Figure S1: Proteomic analysis of hypothalamic tissues; Figure S2: Heat-map hierarchical cluster analysis; Figure S3: Adiponectin level in plasma.
