# Supplementary material for: Fructose Diet-Associated Molecular Alterations in Hypothalamus of Adolescent Rats: A Proteomic Approach
Source: Nutrients. 2023 Jan 16;15(2):475. doi: 10.3390/nu15020475 (PMC9862284; doi:10.3390/nu15020475)
Supplement: Supplementary file 1 [file nutrients-15-00475-s001.zip › nutrients-2133455-supplementary.pdf]

Fructose diet-associated molecular alterations in hypothalamus of adolescent rats: a proteomic approach

Chiara D'Ambrosio[1†], Luisa Cigliano[2†], Arianna Mazzoli[2], Monica Matuozzo[1], Martina Nazzaro[2], Andrea Scaloni[1], Susanna Iossa[2] and Maria Stefania Spagnuolo[1*]

[1]Institute for the Animal Production System in the Mediterranean Environment, National Research Council, 80055 Portici (Naples), Italy.

[2]Department of Biology, University of Naples Federico II, 80121 Naples, Italy.

[†] These authors contributed to equally to this work

*Correspondence:
Maria Stefania Spagnuolo, Institute for the Animal Production System in the Mediterranean Environment, National Research Council, Piazzale Enrico Fermi 1, 80055 Portici (NA), Italy.
E-mail: mariastefania.spagnuolo@cnr.it

SUPPLEMENTARY MATERIAL

Table S1. Identification details of differentially represented proteins

Reported are the information on protein false discovery rate (FDR) confidence, protein accession, description, gene name, exp. q-value, sum posterior error probability (PEP) score, sequence coverage (%), number of identified peptides, peptide spectrum matches (PSMs), number of identified unique peptides, number of amino acids, molecular mass, pI, Mascot identification score values, Razor Peptides, Abundance Ratio, Abundance Ratio (log2), Abundance Ratio P-Value, Abundance Ratio Adj. P-Value, Abundances (Grouped), Abundances (Grouped) CV [%].

FDR Confidence: Combined	Accession	Description	Gene name	Exp. q-value: Combined	Sum PEP Score	Coverage [%]	# Peptides	# PSMs	# Unique Peptides	# AAs	MW [kDa]	calc. pI	Mascot: Mascot	Peptides (by Search Engine):	# Razor Peptides	Abundance Ratio: F/C	Abundance Ratio: FR / CR	Abundance Ratio (log2): F/C	Abundance Ratio (log2): FR / CR)	Abundance Ratio P-Value: F / C	Abundance Ratio P-Value: FR / CR	Abundance Ratio Adj. P-Value: F / C	Abundance Ratio Adj. P-Value: FR / CR	Abundances (Grouped): C	Abundances (Grouped): CR	Abundances (Grouped): F	Abundances (Grouped): FR	Abundances (Grouped) CV [%]: C	Abundances (Grouped) CV [%]: CR	Abundances (Grouped) CV [%]: F	Abundances (Grouped) CV [%]: FR
High	D4AEC	Histone H2A OS=Rattus norvegicu	H2afv	0	7,211	15	2	7	1	128	13,5	10,58	137	2	0	1,663	1,153	0,73	0,21	0,000144367	0,103786106	0,098440031	1	78,3	88,9	130,3	102,5	7,7	4,49	7,77	2,82
High	P01186	Vasopressin-neurophysin 2-copept	Avp	0	32,91	46	5	17	5	168	17,9	6,42	402	5	0	1,253	0,999	0,33	0	0,017325092	0,999878548	1	1	88,9	100	111,3	99,8	3,9	2,56	1,48	2,22
High	P04631	Protein S100-B OS=Rattus norveg	S100b	0	47,209	40	4	98	4	92	10,7	4,55	2444	4	0	1,25	0,908	0,32	-0,14	0,022714908	0,362054366	1	1	79,4	115,9	99,3	105,3	1,36	0,94	0,74	1,28
High	G3V9G	Calcium/calmodulin-dependent pro	Camk2b	0	106,09	44	17	81	9	542	60,4	7,28	2077	17	8	0,828	0,938	-0,27	-0,09	0,042986627	0,667514111	1	1	104	108,3	86,1	101,6	0,96	1,05	0,58	0,73
High	D3ZW0	Sortilin-related VPS10 domain-con	Sorcs2	0	5,603	2	2	2	2	1158	128,7	7,37	46	2	0	0,812	1,033	-0,3	0,05	0,011712893	0,779640991	1	1	110,1	98,7	89,4	101,9	3,99	5,18	0,89	0,07
High	Q66HF1	NADH-ubiquinone oxidoreductase	Ndufs1	0	160,73	50	25	105	25	727	79,4	5,9	2687	25	0	0,792	0,904	-0,34	-0,15	3,59956E-06	0,002126498	0,01631957	1	100,9	115,1	79,9	104,1	1,1	0,9	1,73	0,46
High	G3V8F1	Transporter OS=Rattus norvegicus	Slc6a7	0	6,307	3	2	5	2	637	71	6,61	116	2	0	0,787	1,035	-0,35	0,05	0,009751426	0,792525536	1	1	116,2	94,6	91,4	97,8	2,67	0,7	6,43	2,17
High	B5DEH	Erlin-2 OS=Rattus norvegicus OX	Erlin2	0	22,374	17	6	12	6	339	37,7	5,74	174	6	0	0,787	0,929	-0,35	-0,11	0,032382357	0,661928666	1	1	112,8	102,9	88,7	95,6	1,79	1	2,89	0,54
High	M0RCX	4a-hydroxytetrahydrobiopterin deh	Pcbd2	0,001	4,508	11	2	5	1	127	14,1	9,2	68	2	0	0,778	0,92	-0,36	-0,12	0,040529395	0,646961752	1	1	104,9	111,2	81,6	102,3	1,35	8,09	9,64	0,79
High	D3Z9R8	ATP synthase subunit ATP5MJ, m	Atp5mj	0	6,537	30	2	4	2	60	6,9	9,92	65	2	0	0,776	0,969	-0,37	-0,05	0,000198956	0,247206181	0,120589251	1	99,1	113,8	76,9	110,2	0,86	1,01	1,89	1,59
High	P19527	Neurofilament light polypeptide O	Nefl	0	134,43	55	26	152	23	542	61,3	4,65	2913	26	0	0,761	0,907	-0,39	-0,14	0,001265901	0,191562101	0,531191734	1	99,1	118,2	75,4	107,2	1,25	1,99	2,88	0,92
High	B2RYW	Complex I-B22 OS=Rattus norveg	Ndufb9	0	7,726	20	3	12	3	179	21,9	8,07	301	3	0	0,737	0,903	-0,44	-0,15	5,9624E-05	0,03306631	0,070895365	1	99,6	119,3	73,5	107,7	1,58	2,07	1,46	1,36
High	D3ZD0	Cytochrome c oxidase subunit OS=	Cox6b1	0	20,381	62	5	24	5	86	10,1	8,72	574	5	0	0,729	0,913	-0,46	-0,13	3,43772E-05	0,132018961	0,062509183	1	98,3	120,2	71,6	109,8	1,67	1,53	6,13	2,06
High	D4A0T	Complex I-PDSW OS=Rattus norv	Ndufb10	0	21,078	26	4	16	4	176	20,8	7,69	370	4	0	0,726	0,886	-0,46	-0,17	0,019276616	0,425969694	1	1	101,8	118,9	73,9	105,3	0,53	1,4	5,21	1,66
High	P12839	Neurofilament medium polypeptide	Nefm	0	149,86	43	35	153	31	846	95,7	4,79	3047	35	5	0,709	0,903	-0,5	-0,15	0,029305965	0,734079869	1	1	102,4	118,2	72,6	106,8	0,83	0,52	1,88	0,61
High	P23565	Alpha-internexin OS=Rattus norve	Ina	0	145,31	55	27	217	2	505	56,1	5,22	5419	27	21	0,688	0,88	-0,54	-0,18	5,98334E-06	0,009589728	0,01631957	1	99,7	123,3	68,6	108,4	0,85	0,86	1,76	1,01
High	A0A0G	Voltage-dependent anion-selective	Vdac3	0	69,843	54	12	39	11	283	30,8	8,59	1038	12	1	0,563	0,799	-0,83	-0,32	0,012661965	0,4642522	1	1	100,9	134,7	56,8	107,7	1,25	2,29	8,24	1,3
High	Q9Z2L0	Voltage-dependent anion-selective	Vdac1	0	123,83	78	16	100	16	283	30,7	8,54	2838	16	0	0,561	0,819	-0,83	-0,29	6,4982E-05	0,052250196	0,070895365	1	103,2	131,4	57,9	107,6	1,46	0,98	4,97	0,25
High	P81155	Voltage-dependent anion-selective	Vdac2	0	64,382	52	11	51	10	295	31,7	7,49	1111	11	0	0,534	0,816	-0,9	-0,29	0,005847761	0,455456088	1	1	102,1	133,9	54,6	109,4	0,72	1,77	7,31	1
High	A1A5N	Hyaluronan and proteoglycan link	Hapln1	0	9,701	8	2	6	2	354	40,2	7,75	190	2	0	0,909	1,306	-0,14	0,39	0,027758747	1,61383E-05	1	0,022008571	96,7	93,4	87,8	122,1	1,99	4,19	0,17	1,67
High	D4AA1	Dmx-like 1 OS=Rattus norvegicus	Dmxl1	0	8,97	1	3	4	1	3017	336,8	6,44	91	3	0	1,105	1,327	0,14	0,41	0,296211629	0,013904761	1	1	90,9	89,6	100,5	119	3,82	2,01	3,26	7,18
High	A0A0G	Transgelin OS=Rattus norvegicus (	Tagln	0	39,171	40	9	20	8	207	23,3	8,66	488	9	0	1,046	1,48	0,07	0,57	0,933241414	0,022808411	1	1	100,2	78,6	104,9	116,3	1,76	3,19	3,51	4,26
High	A0A140	Protein kinase C delta type OS=Ra	Prkcd	0	5,995	3	2	6	1	673	77,5	7,83	97	2	0	0,912	1,281	-0,13	0,36	0,455960047	0,039963264	1	1	108,9	84,1	99,2	107,8	1,17	1,18	0,1	0,36

Figure S1. Proteomic analysis of hypothalamic tissues

Volcano plot representing differentially expressed proteins in hypothalamic tissues isolated from fructose-fed adolescents versus control adolescent rats. Over- and down-represented proteins (Log2FC > 0.26, p < 0.05) are reported in red and green area, respectively.

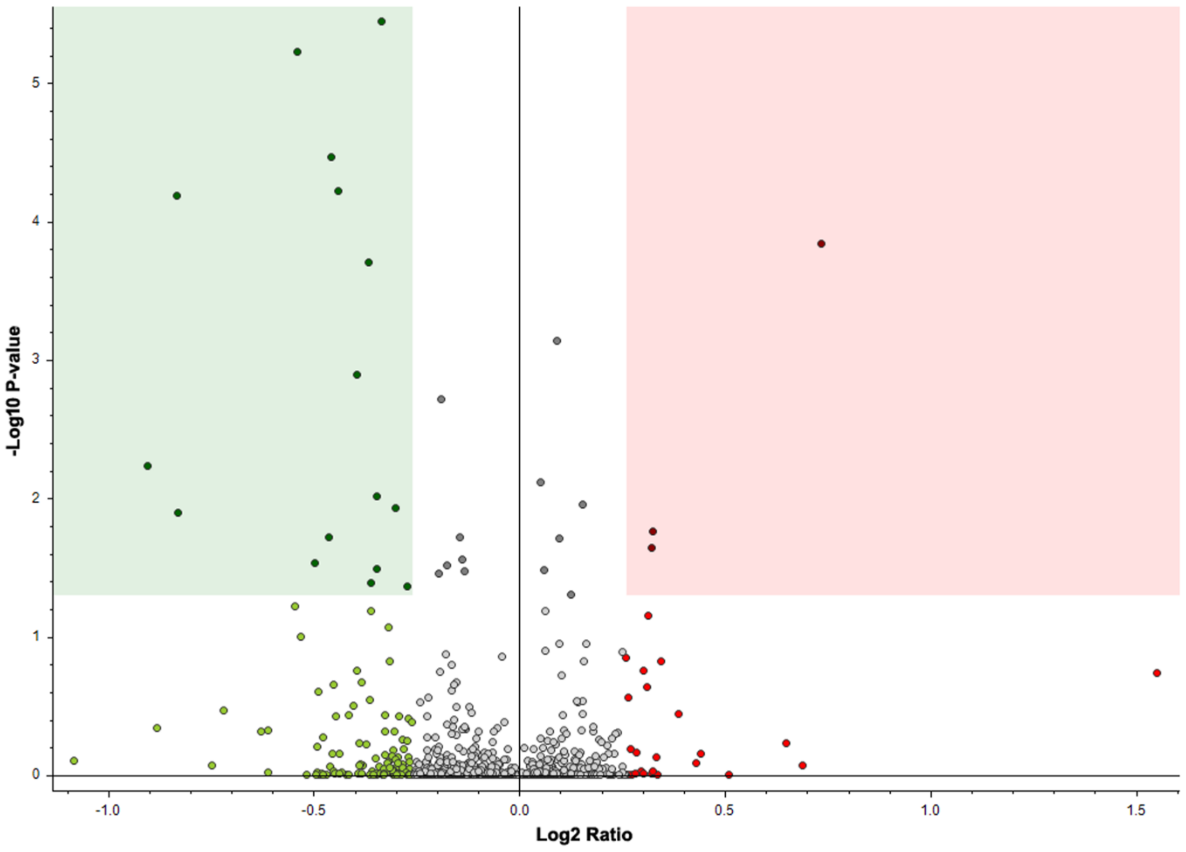

Figure S2. Heat-map hierarchical cluster analysis

Heat-map hierarchical cluster analysis of proteins differentially represented in hypothalamic tissues of fructose-fed adolescent (F, orange) and control adolescent (C, light blue) rats, young-adult fructose-rescued (FR, green) and young-adult control rescued (CR, red) rats. Dendrograms on the left report the protein grouping and distance between protein classes, while dendrograms on the top report the sample grouping. Normalized area ranges and Eculidean distance were used; scaled expression values of each range are plotted in green to red through black color scale.

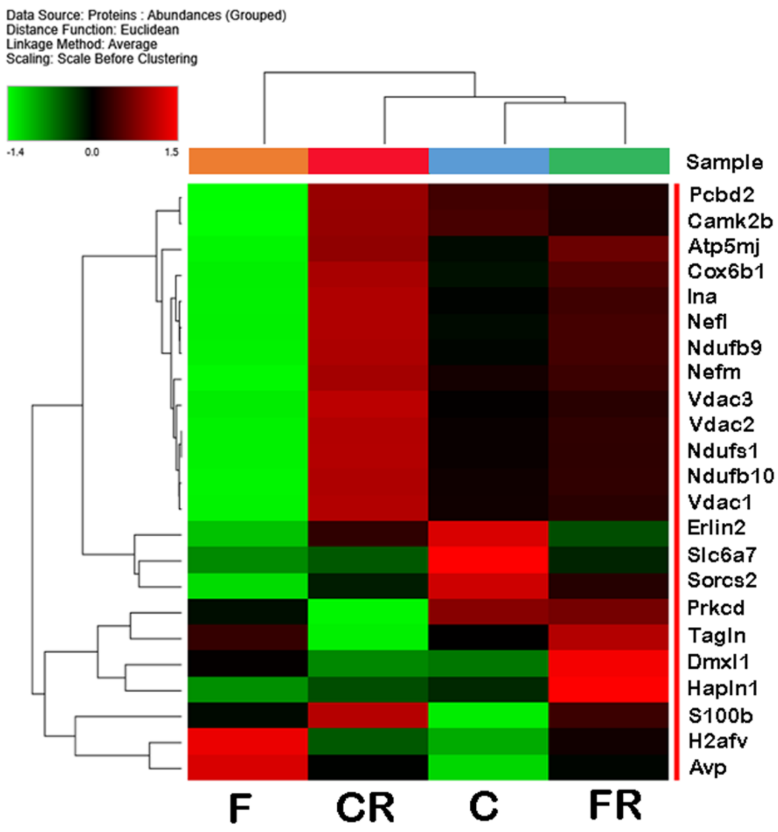

Figure S3. Adiponectin level in plasma

Adiponectin concentration was measured in plasma samples of control adolescent (C), fructose-fed adolescent (F), young-adult control rescued (CR), and young-adult fructose-rescued (FR) rats.

All the plasma samples were adjusted to protein concentration of 10 µg/µL and 5 µL of each sample were analyzed by 12.5 % SDS PAGE and western blotting. Immunocomplexes were detected by rabbit anti-adiponectin and GAR-HRP IgGs. Densitometric data are reported as means ± SEM of 6 different rats from each experimental group. ** $p < 0.01$ vs C; # $p < 0.05$ vs CR (one-way Anova followed by Bonferroni post-test).

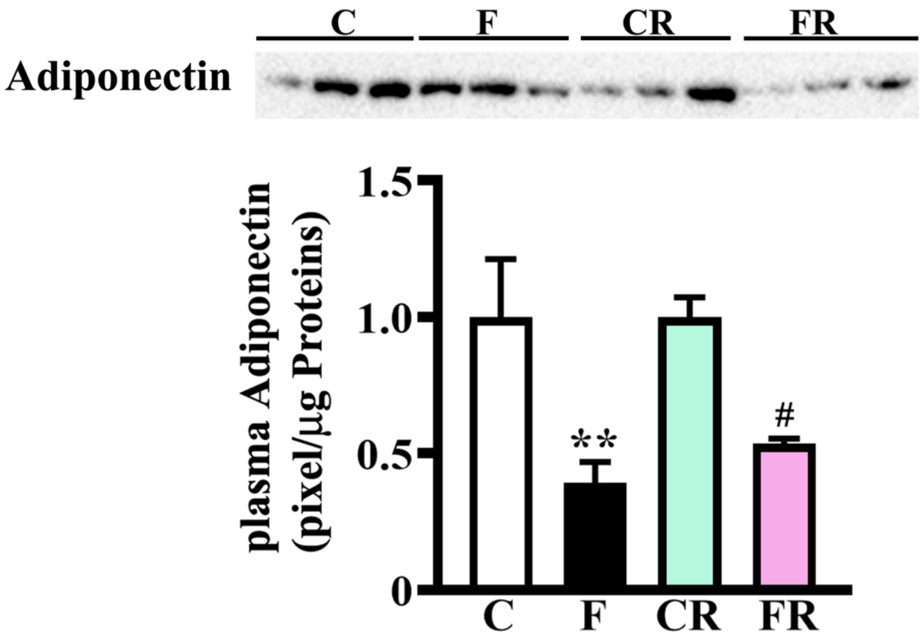

Figure S3
